# Successful management of poisoning with ivermectin (Mectizan) in the Obala health district (Centre Region, Cameroon): a case report

**DOI:** 10.1186/s13256-023-03891-4

**Published:** 2023-04-17

**Authors:** Cyrille Donfo-Azafack, Hugues C. Nana-Djeunga, Guy Wafeu-Sadeu, Raïssa Dongmo-Yemele, Joseph Kamgno

**Affiliations:** 1 Centre for Research on Filariasis and other Tropical Diseases (CRFilMT), PO Box 5797, Yaoundé, Cameroon; 2 Faculty of Medicine and Biomedical Sciences, University of Yaoundé I, Yaoundé, Cameroon

**Keywords:** Ivermectin, Mectizan, Poisoning, Case report, Obala district hospital, Cameroon

## Abstract

**Background:**

Ivermectin (Mectizan) is an anthelmintic drug that plays a major role in the fight against two major filarial diseases, onchocerciasis and lymphatic filariasis. Unlike previous drugs that had serious and sometimes fatal side effects, ivermectin has been considered to be safe and, therefore, is widely used. Data reporting the clinical presentation of poisoning with ivermectin are very scanty, even in experimental studies.

**Case presentation:**

In this paper, we report the case of a 19-year-old Black African female student residing in Obala (Centre Region, Cameroon) who was admitted to a health facility in Central Cameroon for a neurological disorder after intoxication with about 400 tablets of ivermectin 3 mg (~100 times the standard doses). This neurological disorder was characterized by somnolence, kinetic ataxia, increase of tendon reflex, and central visual disturbance. Management of this intoxication consisted of symptomatic treatment and monitoring of hemodynamic parameters for 5 days, with a favorable course.

**Conclusions:**

This is the first report of a poisoning with ivermectin at ~100 times the recommended dose. This case report confirms the safety and tolerability of ivermectin, even at exceptionally high dose.

## Background

Ivermectin is a macrocyclic lactone produced by the actinomycete *Streptomyces avermitilis* [1]. This drug was first used in veterinary medicine until clinical trials revealed a very potent microfilaricidal action in human onchocerciasis [2]. Ivermectin is considered a wonder drug that has transformed the lives of millions of people infected with onchocerciasis since its introduction in human medicine in 1987 [3, 4]. It is active at extremely low doses against a wide variety of parasitic nematodes and arthropods, through its action on the mediation of neurotransmission by gamma-aminobutyric acid [1]. Ivermectin is taken orally as tablets, and a single dosage of 150 µg/kg of body weight administered once a year is highly effective against onchocerciasis [5]. In Africa, onchocerciasis control currently relies on the Community-Directed Treatment with Ivermectin (CDTI) strategy. Except serious adverse reactions that have been reported in Central Africa [6] among patients with *Loa loa* microfilaremia exceeding 30,000 microfilariae (mf) per ml of blood [7], ivermectin is considered as a safe medicine, especially in areas where loiasis is not endemic. Indeed, a systematic review and meta-analysis investigating the safety of high-dose ivermectin confirmed the safety of ivermectin at doses up to 800 µg/kg; the severity of adverse events was mild or moderate for all the studies screened, and was comparable to standard doses (150–200 µg/kg) [8].

In this paper, we are reporting a case of ivermectin poisoning with ~15,000 µg/kg (~100 times the standard dose for onchocerciasis control) that occurred in the Centre Region of Cameroon and was managed by the Obala District Hospital staff with the guidance of the team of the Centre for Research on Filariasis and other Tropical Diseases (CRFilMT).

## Case presentation

A 19-year-old Black female student residing in Obala (Centre Region, Cameroon) in Central Africa, was admitted in the Obala District Hospital on December 12, 2018 with a mildly altered state of consciousness [Glasgow Coma Score (GCS) of 13/15], after consuming a large quantity of ivermectin 3 mg tablets. Indeed, after an argument with her father when returning from school 1 day before admission to the hospital, the patient voluntarily swallowed about 400 ivermectin 3 mg tablets with suicidal intention. She opened a sealed box of 500 tablets of ivermectin 3 mg and when she was found in an altered state of consciousness (GCS 13/15), less than 100 tablets were remaining. About 1–2 hours after ivermectin intake, the patient experienced several episodes of vomiting containing some tablets, asthenia, and a mild altered state of consciousness. The persistence of the symptoms prompted her family to take her to the hospital. On admission, the patient was conscious but presented with an altered general state (asthenia and anorexia), nausea, rotatory vertigo, headache, and visual problems. All her vital signs were normal, but her Glasgow Coma Score was 13/15. Physical examination revealed bilateral mydriasis and reactive pupils, regular heartbeat with a 2/10 functional murmur at the mitral focus, and marked abdominal tenderness at the right iliac fossa. Neurological examination showed a kinetic ataxia with a small increase of tendon reflex. Uncorrected visual acuity was 1/10 for both eyes. Paraclinical examinations consisted of an abdominopelvic ultrasound showing a dilatation of the digestive loops with hyperperistalsis in favor of a spasmodic disorder and epigastric sensitivity at the passage of the probe. An electrocardiogram and electroencephalogram were normal. As for biological assessment, her blood count was normal, uremia and creatinemia were normal, while serum electrolyte test revealed a decrease in potassium concentration at 3.37 Meq/L (3.5–5.4 Meq/L), likely associated with vomiting. The assessment of liver function [transaminase, bilirubin, and gamma-glutamyl transferase (GGT)], the fundus, and the eye scanner were normal. The post-ivermectin *Loa loa* microfilarial density, following a calibrated thick blood smear, was negative.

Of note, a notion of visual pathology was reported in her family, and she reported a visual disturbance prior to the intoxication that never required consultation. The patient also had a past medical history of unconfirmed asthma. She is the third of five siblings with two half-sisters, living with her father and mother-in-law, and had no known psychiatric pathology. She had no known allergy to any drug and had never swallowed ivermectin before this unfortunate event.

Management consisted of saline-based hyper-hydration, antiulcer (omeprazole 40 mg daily) for gastritis, paracetamol 1 g in case of headache, and monitoring of hemodynamic parameters. The evolution was satisfactory from day 2 to day 4 with a decrease of vertigo, asthenia, and ataxia, but a persistent decrease in visual acuity. She was discharged on day 4 despite slight asthenia and visual impairment. One month later, she was psychiatrically stable but presented with persistent visual disturbance. Six months after discharge, she passed the baccalaureate examination and was admitted at the university. She has been diagnosed with, and is currently treated for, myopia and hyperopia, likely present a while before the intoxication event.

## Discussion and conclusions

Data reporting ivermectin poisoning in humans are very scanty. It was previously demonstrated that ivermectin is well tolerated at doses up to ten times the highest Food and Drug Administration (FDA)-approved dose (200 µg/kg), with no evidence of associated toxicity, indicating that ivermectin accumulation is minimal [9]. Another study involving 12 healthy volunteers who each received a single oral dose of 12 mg of ivermectin confirmed this hypothesis [10]. In the present report, however, the hypothetical dose of ivermectin 3 mg ingested (~1200 mg) was approximately 100 times higher than the highest recommended dose. This high dose of ivermectin resulted in poisoning involving a central nervous system depression with dizziness, ataxia, headache, drowsiness, mydriasis, and a mildly altered state of consciousness (GCS, 13/15). A previous experimental study in rats revealed that gradually increased doses of ivermectin were associated with drowsiness at 10 mg/kg and depression of the central nervous system from 15 mg/kg [11], though these symptoms were reversible after a few days. Our patient presented with bilateral mydriasis, kinetic ataxia, and hyperreflexia. These signs could be due to ivermectin stimulation of the orthosympathetic nervous system, resulting in bilateral mydriasis, and the parasympathetic nervous system, leading to an exacerbation of tendon reflex. Indeed, an experimental study in calves has previously demonstrated that some clinical signs such as ataxia are due to the cholinergic function of ivermectin mediated by gamma aminobutyric acid (GABA). Similarly, mydriasis and ataxia were also observed in some dogs receiving up to 600 mg/kg of ivermectin administered subcutaneously [12]. In a double-blind placebo-controlled study, doses were higher and/or more frequent than those currently approved for human treatment, and the primary safety endpoint was mydriasis, accurately quantified by pupillometry [9]. In another double-blind placebo-controlled trial, some patients treated with 800 µg/kg experienced a subjective dyschromatopsia or colored vision (yellow or red) [13]. Our patient experienced reduced visual acuity, but this should be taken with caution since the patient reported vision problems before ivermectin poisoning. Also, the inability of ivermectin to cross the aqueous humoral barrier was previously described, likely due to its high molecular size, as other macrocyclic lactones. The blood-eye barriers thus block the penetration of ivermectin into the ocular globe [14]. An MRI would have been necessary to better investigate the causes of this visual deficit. A blood test of ivermectin would also have been interesting to confirm the poisoning, as well as to monitor the elimination of the active principle.

This is the first report of a poisoning with ivermectin of ~100 times the recommended dose. This case report confirms the safety and tolerability of ivermectin, even at exceptionally high doses.

## Data Availability

All data generated or analysed during this study are included in this published article.

## Abbreviations

CDTI: Community-directed treatment with ivermectin
FDA: Food and Drug Administration
GABA: Gamma aminobutyric acid
IVM: Ivermectin
MRI: Magnetic resonance imaging
WHO: World Health Organization
